# A Case of Fungemia Caused by Postoperative Chronic Lumbar Intervertebral Disc Infection

**DOI:** 10.1155/2022/8311278

**Published:** 2022-08-08

**Authors:** Rui Zheng, Taicheng Jin, Rui Tao, Ning Xu

**Affiliations:** ^1^Department of Clinical Laboratory, Affiliated Hospital of Kunming University of Science and Technology, Xishan, Kunming, China 650032; ^2^Department of Clinical Laboratory, The First People's Hospital of Yunnan Province, 157 Jinbi Rd, Xishan, Kunming, Yunnan, China 650021; ^3^Hamilton College, 198 College Hill Rd, Clinton, NY 13323, USA

## Abstract

Postoperative surgical site infection is one of the serious postoperative complications of spine surgery, especially fungal infections. Late-stage surgical site fungal infections often lack typical clinical symptoms and have a variable clinical presentation. In this case, the patient was a senior patient with usually only tolerable pain and discomfort, which was detected 2 years after the first surgery. Such cases are even rarer for fungal bloodstream infections caused by delayed postoperative chronic fungal osteomyelitis and deserve further study for early identification and intervention to minimize harm.

## 1. Introduction

Infection is one of the thorniest clinical problems in orthopedics, especially spinal infections, which can prolong the course of the disease and lead to catastrophic consequences in severe cases. Spinal fungal infection and spinal tuberculosis belong to granulomatous inflammation, which is difficult to distinguish from each other in the early stage. Diagnostic and detective methods' low sensitivity and specificity can lead to delayed diagnosis and adversely impact prognosis [1]. Spinal fungal osteomyelitis is a rare case of fungal infection, usually caused by Aspergillus or Candida [2]. Since postspinal surgery fungal bloodstream infections is rarer, we report here a case of chronic lumbar intervertebral disc infection that leads to fungemia after secondary spinal repair. Lessons to be learned from this report include (1) the relevance of considering changes in blood test results in cases of spinal fungal infections with scant clinical symptoms and (2) the usefulness of the relatively new testing method, MALDI-TOF MS.

## 2. Case Presentation

**Figure 1:**
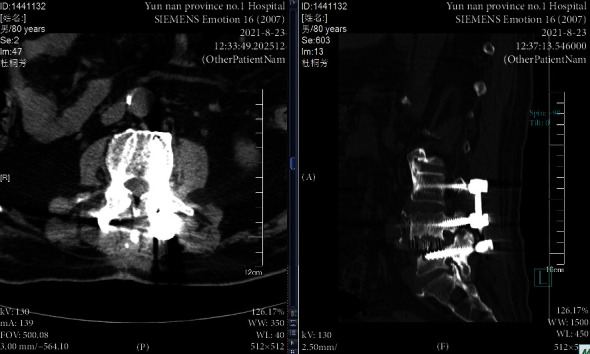
Preoperative CT imaging of the fourth and fifth lumbar spine.

**Figure 2:**
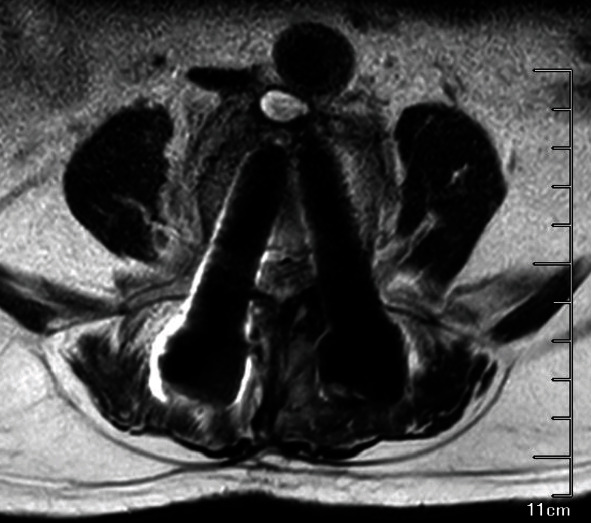
Preoperative MRI of the fourth and fifth lumbar spine.

**Figure 3:**
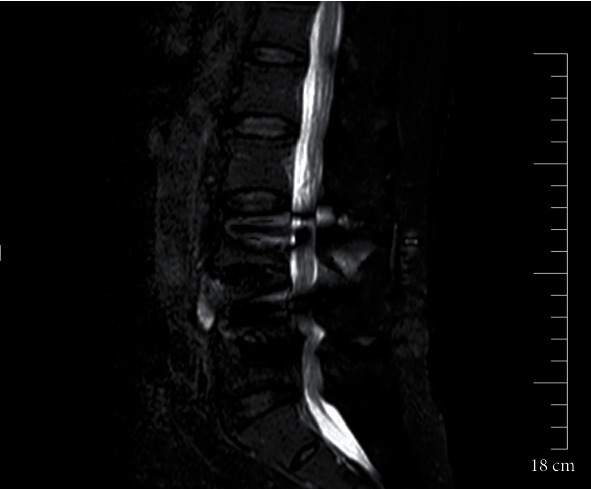
Preoperative MRI of the fourth and fifth lumbar spine.

An 80-year-old male underwent lumbar internal fixation due to lumbar spinal stenosis. A year and a half after surgery, the patient presented to the local county hospital in April of 2021 with low back pain with bilateral lower extremities radiating pain for 2 weeks, no signs of spinal cord injury on examination, CT images were lost, medical records described a soft tissue edema signal around the caudal part of the L4 right cone arch screw, no fever, laboratory tests, normal blood count, PCT 0.5 ng/mL, CRP 36.2 mg/L, ESR 60 mm/h, negative blood culture, still considering infection, cefuroxime was given. Discharged after 5 days of treatment, symptoms persisted. 16 weeks later, the patient returned to the hospital with pain and discomfort in both lower limbs. 1 week before the return visit, the patient developed severe lumbosacral pain with no obvious cause, independent of weather changes. On admission, the radiographs showed a poorly defined lumbar 4-5 space and a slightly compressed dural sac. CT films showed a short dense shadow in the lumbar 4-5 space (Figure 1). The MRI findings confirmed the disorganization of the spinal fixation device and screw fractures and a short strip of dense shadow in the lumbar 4-5 space, which was suspected to be chronic osteomyelitis (Figures 2 and 3). Anterolateral approach lumbar discectomy was performed to remove the interbody fusion and posterior lumbar interbody fixation by the nail-stick system. During the operation, the L4-5 intervertebral tissue showed scattered light-yellow residue-like pus. After the necrotic tissue, purulence, bone lesions, and surrounding granulation tissue are removed; the affected segment was stabilized and fused with the reconstructed segment and washed with a large amount of saline. The incision area was given iodophor and the incision was washed with normal saline. The inflammatory lesion was taken for examination and tissue culture. On the second day, the patient presented with irritability, with a body temperature of 37.9°C, a heart rate of 106 bpm, white blood cell count (WBC) of 9.63 × 10^9^/L, neutrophilic granulocyte percentage (NGP) 78.5%, lymphocytes percentage (LP) 16.7%, and C-reactive protein (CRP) 47.20 mg/L, interleukin-6 36.50 pg/mL, and procalcitonin 4.13 ng/mL. Ceftriaxone was immediately prescribed. Direct smear microscopy of the stent pus showed yeast-like fungal spores and hyphae, and blood was drawn for (*β*-D-glucan test, 165 *μ*g/L). Bone marrow and blood cultures (aerobic and anaerobic) were sent for pathology at the same time, and the pathology report showed local tissue necrosis and fungal hyphae. The culture were inoculated in Columbia Blood Agar Base (Autobio, China), respectively, at 35°C, 5% CO_2_ incubator. On the 3rd day, MALDI-TOF MS (bioMérieux, Marcy l'Étoile, France) identified Candida albicans (99% CI (LL, UL)) with the calibration strain and quality control strain ATCC MYA-2950. Ceftriaxone treatment was stopped clinically and changed to 0.4 g of fluconazole for 12 hours. From the 5th day onwards, no microorganisms were found in the blood culture. After 10 days of treatment, the patient's body temperature and various inflammatory indexes were normal, and the general condition was stable, so he was discharged from the hospital. Patient was administered oral fluconazole for 2 weeks and recovered.

## 3. Discussion

Candida albicans is ubiquitous in nature and is a conditional pathogenic fungus. In 1932, Keating et al. first reported spinal fungal infections. Spinal fungal infections are systemic infections, and the recurrence rate and mortality rate are higher than general bacterial infections [3]. When the host has susceptibility factors for fungal infection and abuse of antibacterial drugs, it will mostly cause respiratory, digestive, and genitourinary tract infections. With current detection methods, low specificity is prone to delay in diagnosis and results in high rates of missed diagnosis and misdiagnosis. It is rare for the fungal infection to invade the spine [4]. Fungal infection of the intervertebral space that leads to fungemia after the second repair surgery is even rarer. There are many risk factors for spinal fungal infection, which are generally related to immunosuppression. Related underlying diseases or pathogenic conditions include diabetes, glucocorticoid use, chemotherapy and malnutrition, AIDS, organ transplantation, and intravenous drug use [5]. The patient's atypical medical history and clinical examination are often the cause of delayed diagnosis. In this case, the infection progressed very slowly and lasted for nearly a year. The patient was an elderly diabetic patient with long-term pain in both lower limbs after the operation. Imaging manifestations of fungal infections are obtained. Postoperative fungemia is closely associated with the patient's advanced age and diabetes [6, 7]. The study by Quindós [8] showed that mycelia in the elderly lack typical symptoms of bacteremia. Most of the symptoms of systemic poisoning in osteomyelitis are mild and local acute inflammation comes slowly, such as fever, chills, and increase in white blood cells, It is easy to cause clinicians' neglect in the early stage, which leads to high mortality. Early diagnosis and early treatment are essential to control candidiasis and prevent complications. In recent years, matrix-assisted laser desorption ionization time of flight mass spectrometry (MALDI-TOF MS) has compensated for the disadvantages of traditional methods with higher accuracy and faster processing time [9–11]. MALDI-TOF MS technology enables accurate and quick identification of obscure fungi species and facilitates the correct application of antifungal drugs, averting disastrous consequences for this elderly diabetic patient. In addition, because elderly patients with underlying diseases have complex and combination conditions and are prone to various drug-related problems, the role of clinical pharmacists warrants additional attention in the joint formulation of the best treatment plan, which will help improve the success rate of clinical treatment and improve the prognosis of patients.

## 4. Conclusion

This case shows the potential of postspinal surgery lumbar pain and discomfort in both lower extremities as signs of chronic spinal infection, especially for elderly patients with underlying diseases. Therefore, the possibility of fungal infection should be considered clinically in immunocompromised elderly patients with underlying disease to prevent underdiagnosis and misdiagnosis.
